# Supplementary material for: Safety and Efficacy upon Infection in Sheep with Rift Valley Fever Virus ZH548-rA2, a Triple Mutant Rescued Virus
Source: Viruses. 2024 Jan 5;16(1):87. doi: 10.3390/v16010087 (PMC10819402; doi:10.3390/v16010087)
Supplement: Supplementary file 1 [file viruses-16-00087-s001.zip › viruses-2809654-supplementary.pdf]

IFNg ELISA

	sheep #			
	39	40	768	909
d0	0,053	0,101	n.d	n.d
d2	0,053	0,099	n.d	n.d
d4	0,096	0,174	n.d	n.d
d7	0,079	0,115	n.d	n.d
d14	0,051	0,09	n.d	n.d
d21	0,05	0,09	n.d	n.d
d1 postch	0,06	0,13	0,227	0,251
d2 postch	0,07	0,171	0,07	0,214
d3 postch	0,104	0,179	0,221	0,747
d4 postch	0,043	0,1	n.a	0,923

Biochemistry

	sheep #			
**AST**	909	768	39	40
0	348	264	82	111
1	277	457	87	94
2	269	380	91	98
3	260	330	87	92
4	282	n.a	n.a	95

	sheep #			
**BUN**	909	768	39	40
0	21	20	15	15
1	19	28	25	22
2	22	27	30	29
3	21	29	26	21
4	23			22

Temperatures

	sheep #			
days post-inoculat	39	40	73	83
-3	39,8	39,5	40	40,2
-2	39,9	40,3	40	39,7
-1	39,3	39,4	39,9	39,1
0	40,2	39,7	39,9	39,8
1	40,3	39,9	40,2	40,3
2	40	39,8	40	39,9
3	39,8	39,8	39,9	39,5
4	40	39,4	39,2	39,8
5	40,3	39,6	39,8	39,4
6	40	39,9	39,8	39,5
7	39,7	39,4	40	39,7

restimulation proteins

	d7		d14		d2
sheep #	39	40	39	40	39
	0,255	0,084	0,184	0,404	0,908
	0,127	0,177	0,213	0,203	1
	0,129	0,077	0,083	0,107	0,118

SNT

day	39	40	73	83
	sheep #			
2	1,477121	1,30103		
3	1,60206	1,30103		
4	2	1,69897		
5	2,30103	2		
7	2,30103	2	2,60206	2,30103
14	2,60206	2,30103		
21	2,60206	2,30103		
post-challenge	3,50515	3,50515		

	sheep #			
**GGT**	909	768	39	40
0	62	55	48	85
1	64	61	58	72
2	57	58	62	78
3	59	53	54	71
4	63			73

	sheep #			
**ALB**	909	768	39	40
0	3,9	3,3	4	3,6
1	4,1	3,9	3,7	4
2	4,2	3,7	3,8	4,2
3	4,1	3,5	3,6	4
4	4			4

	sheep #			
days post challer	909	768	39	40
-5	39,2	39,6	39,6	39,2
-4	39,4	39	39,6	39,1
-3	39	39,2	39,6	39
-2	39,2	39,3	39,5	39,2
-1	39,1	39,3	39,5	39
0	39,8	39,1	39,3	39,1
1	39,6	39,7	39,7	39,3
2	39,9	40,1	39,4	39,3
3	40,4	39,9	39,5	39,3
4	41,3			39,1

	**restimulation peptides**				
21		d7		d14	
40	sheep #	39	40	39	40
0,764		0,097	0,085	0,119	0,082
0,509		0,146	0,132	0,13	0,906
0,1		0,12	0,125	0,12	0,127
		0,131	0,089	0,115	0,124
		0,156	0,189	0,288	0,133

d21	
39	40
0,157	0,182
0,337	0,665
0,502	1
0,606	0,277
1	1
